# Technical Procedures for Preparation and Administration of Platelet-Rich Plasma and Related Products: A Scoping Review

**DOI:** 10.3389/fcell.2020.598816

**Published:** 2020-12-11

**Authors:** Daniela Vianna Pachito, Ângela Maria Bagattini, Adriano Marques de Almeida, Alfredo Mendrone-Júnior, Rachel Riera

**Affiliations:** ^1^Núcleo de Avaliação de Tecnologias em Saúde, Hospital Sírio-Libanês, São Paulo, Brazil; ^2^Fundação Getúlio Vargas, São Paulo, Brazil; ^3^Instituto de Patologia Tropical e Saúde Pública, Universidade Federal de Goiás, Goiânia, Brazil; ^4^Instituto de Ortopedia e Traumatologia, Hospital das Clínicas, Universidade de São Paulo, São Paulo, Brazil; ^5^Fundação Pro Sangue Hemocentro de São Paulo e Laboratório de Processamento Celular, Hospital Sírio Libanês, São Paulo, Brazil; ^6^Disciplina de Medicina Baseada em Evidências, Escola Paulista de Medicina, Universidade Federal de São Paulo, São Paulo, Brazil

**Keywords:** platelet-rich plasma, platelet-rich fibrin, platelet concentrates, growth factors, platelets

## Abstract

**Introduction:**

Platelet-rich plasma is widely used for different types of clinical situations, but universal standardization of procedures for its preparation is still lacking.

**Methods:**

Scoping review of comparative studies that have assessed at least two alternatives in one or more stages of preparation, storage and/or administration of PRP or its related products. A systematic search was conducted in MEDLINE, Embase, and LILACS. Two authors screened references independently. Data extraction was performed iteratively, and results were presented for each included comparison.

**Results:**

Thirty-nine studies were included after assessing full texts, focusing on the comparison of PRP to a related product, types of anticoagulants, centrifugation protocols, commercial kits, processing time, methods for activation, and application concomitantly to other substances. Only laboratory outcomes were assessed, as platelet, leukocyte and growth factor concentrations.

**Conclusion:**

Results showed great variability related to methods employed in different stages of PRP processing, which may explain the variability observed in clinical trials assessing the efficacy of PRP for different clinical situations.

## Introduction

Platelet-rich plasma (PRP) has been advocated as a therapeutic option for a vast array of clinical situations in different fields of Medicine and Dentistry (Albanese et al., 2013; Robins, 2017). The therapeutic effects of PRP have been attributed to the supraphysiological concentration of growth factors and cell adhesion molecules (Marx, 2004), ultimately leading to, among other effects, angiogenesis, cell proliferation, deposition of collagen, and mesenchymal stem cell differentiation (Smyth et al., 2013).

Despite the increasing demonstration of its efficacy by previous research (Roselló-Camps et al., 2015; Martinez-Zapata et al., 2016; Sadabad et al., 2016; Li et al., 2017), there is still considerable uncertainty about the characteristics of PRP that may lead to optimal results. Clear recommendations about the ideal concentration of platelets and growth factors are still lacking, although a number of studies suggest a dose-effect relation with a ceiling effect (Giusti et al., 2009). Additionally, other characteristics of PRP still remain object of debate, such as the benefits related to white blood cells in PRP (L-PRP) (Bielecki et al., 2012; Wang et al., 2018b).

The variability of procedures applied for preparing PRP and other related products, including plasma-rich fibrin (PRF), along all stages of preparation, such as centrifugation, activation and types of anticoagulants, challenges a uniform recommendation of standardized procedures (Russell et al., 2013). Different terminologies and classification schemes have been proposed to embrace the diversity of procedures for the preparation of PRP (Ehrenfest et al., 2010, 2014; DeLong et al., 2012; Magalon et al., 2016; Lana et al., 2017), as well as frameworks to allow discrimination and specification of processing quantitative and qualitative standards (Gentile et al., 2020).

From the clinical perspective, the lack of standardization hampers the comparation of results from clinical trials that may had employed different protocols for PRP production. This fact may explain the heterogeneity of results observed in these trials and contributes to the uncertainties related to the clinical effects of PRP (Vos et al., 2014). Additionally, the diversity of methods embedded in PRP preparation defies the delineation of regulatory norms, which, by its turn, may contribute to the permissiveness toward substandard practices.

The objectives of this scoping review were to identify and summarize methods employed for preparation, storage and administration of PRP and its related products, and to identify the gaps of knowledge, following an evidence-based approach.

## Materials and Methods

### Study Design

This scoping review was developed in five stages, namely (i) definition of the research question, (ii) elaboration of search strategies, (iii) assessment of study eligibility, (iv) data extraction, and (v) summary of findings. This methodological framework was first proposed by Arksey (Arksey and Malley, 2005) and later revised by Levac (Levac et al., 2010). The study report was structured in a way to contemplate all items of the PRISMA extension for scoping reviews (PRISMA-ScR) (Tricco et al., 2018). A protocol describing the review methods was *a priori* developed and made available at Open Science Framework (doi: 10.17605/OSF.IO/3WZEP).

### Definition of the Research Questions

Research questions were prospectively defined to reflect the aspects susceptible to variability during preparation, storage and administration of PRP. Research questions are presented in Box 1. These questions were iteratively expanded during the stage of data extraction.

**Box 1. Predefined research questions.**

• Methods for obtaining PRP (e.g., open systems, closed systems).

• Activation methods.

• Centrifugation protocols.

• Methods applied for quality control.

### Search Strategies

Search strategies were applied in MEDLINE (via PubMed), Embase (via Elsevier) and LILACS – (via Biblioteca Virtual em Saúde, BVS), on 23rd November 2018 (Supplementary Material). Additionally, reference lists of included studies were hand searched aiming at identifying potentially eligible studies.

### Eligibility Criteria, Study Screening and Data Extraction

Inclusion and exclusion criteria were iteratively defined along data extraction, as previously recommended by Arksey and Levac (Arksey and Malley, 2005; Levac et al., 2010; Table 1). Different types of primary study designs, such as randomized controlled trials, non-randomized trials and *in vitro* studies were considered for inclusion, provided they had assessed at least two alternatives for any stage of preparation of PRP. Studies conducted before 2000 were not considered for inclusion, given that they might not reflect the current standard of practice due to the fast evolution of the field along the last two decades.

**TABLE 1:** Inclusion and exclusion criteria.

**Inclusion criteria**	**Exclusion criteria**
• Comparative research that have assessed at least two alternatives in one or more stages of preparation, storage and/or administration of PRP or its related products.	• Studies published in languages other than English, Spanish and Portuguese. • Studies conducted before 2000. • Secondary studies (e.g., systematic reviews, narrative reviews). • Non-comparative studies. • Comparative studies in which quantitative analyzes were not reported. • Animal studies. • PRP for transfusion. • Studies enrolling participants under antiplatelet therapy. • Studies comparing platelet concentrates to other types of blood components.

Screening of studies was performed at two stages. At the first stage, titles and abstracts were screened independently by two authors, with resolution of disagreements by consensus. At the second stage, full texts were assessed and confronted against the inclusion and exclusion criteria, as they were iteratively defined. Both stages of study screening were conducted in the Rayyan platform (Ouzzani et al., 2016).

### Data Extraction

Data extraction was performed in a Microsoft Excel^®^ spreadsheet (2016). The framework for data extraction was *a priori* defined in a way to reflect the research questions. The final framework was achieved after incorporating relevant aspects.

### Summary of Findings

Study screening was documented and presented in a PRISMA flow diagram. Results were presented narratively, grouped by stage of preparation and administration of PRP.

## Results

**FIGURE 1:**
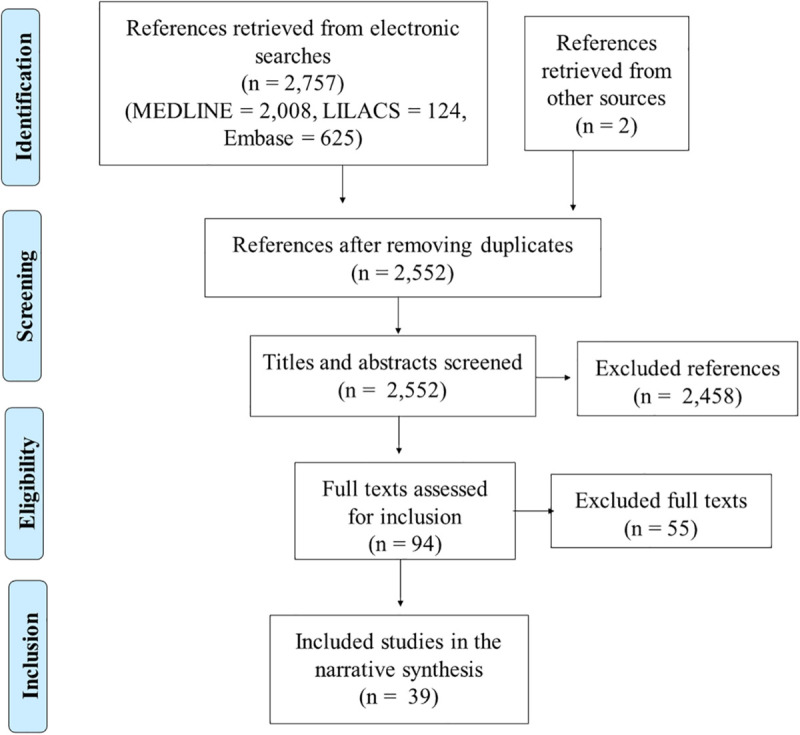
Study flow diagram.

Electronic searches retrieved 2,757 references. Two additional references were additionally identified. After removing duplicates, titles and abstracts of 2,552 references were screened, leading to a selection of 94 studies. Thirty-nine studies were included after the assessment of full texts (Figure 1). The list of excluded studies at the full text stage and reasons for exclusion are presented in Supplementary Material.

### Characteristics of Included Studies

#### Study Design

One non-randomized clinical trial was included (Alhumaidan et al., 2011). The other included studies were *in vitro* controlled studies employing different methods for any one of stage of production of PRP or related products.

#### Research Questions

Included studies addressed different research questions, namely (i) comparison of PRP to related products; (ii) different commercial kits for PRP processing; (iii) types of anticoagulants; (iv) centrifugation protocols; (v) time for PRP processing; (vi) activation methods; and (vii) combined use with other substances.

#### Comparisons of Included Studies

##### PRP compared to other platelet concentrates

Five studies compared the characteristics of PRP to other platelet concentrates (Lachert et al., 2011; Cavallo et al., 2014; Kobayashi et al., 2015; Mariani et al., 2015; Kieb et al., 2017). These studies compared PRP with PRP with leukocytes (L-PRP) (Cavallo et al., 2014; Mariani et al., 2015); powdered PRP (Kieb et al., 2017); and PRF (Kobayashi et al., 2015).

In the study by Cavallo et al. (2014), PRP was compared to L-PRP, regarding the potential of inducing *in vitro* chondrocyte proliferation, concentration of growth factors (GFs) and production of cartilage matrix. L-PRP was demonstrated to present higher concentration of GFs. Both types of platelet concentrate induced chondrocyte proliferation, however PRP was associated with more expressive cell proliferation after seven days of cell culture. PRP-L induced higher levels of genic expression of hyaluronic acid synthase-2.

Mariani et al. (2015) compared PRP to L-PRP in relation to antimicrobial properties, after incubation assays with different pathogens. Both types of platelet concentrate inhibited bacterial growth during a four-hour period of incubation.

Kieb et al. (2017) compared PRP to PRP powder. PRP powder was obtained after sequential stages of depuration of cell components, leading to protein concentration of 30 g/ml. Platelet, leukocyte and GF concentrations were assessed. Both PRP and PRP powder presented higher concentration of GFs (VEGF, bFGF, PDGF-AB and TGF-b1), when compared to whole blood. PRP powder showed higher concentrations of these GF, when compared to PRP.

In the study by Kobayashi et al., PRF was compared to PRP in relation to concentration of GF and the angiogenic and healing effects (Kobayashi et al., 2015). Higher concentration of PDGF-BB was observed in PRP. Results for angiogenic GFs (VEGF and DLL1) were deemed inconsistent. The scratch assay showed better responses of healing for PRF. Similarly, PRF was considered superior regarding neovascularization.

Xian et al. (2015) compared the potential to induce keratinocyte and fibroblast differentiation for PRP with different concentrations (10% PRP and 20% PRP). Concentrations of GFs, cell viability and responses to the scratch assay were also assessed. Higher concentrations of HGF and VEGF-a were found in 10% PRP. Other GFs were not detected in neither of the two groups. Cell cultures with 10% PRP presented more abundant keratinocyte proliferation, however, cultures with 20% PRP showed more collagen fibers types I and III.

##### Comparisons between PRP with different GFs and platelet concentrations

Han et al. (2007) compared PRP with different concentrations of TGF-β1 and PDGF, by assessing its potential to induce proliferation of periodontal ligament cells. The effects on cell proliferation and differentiation occurred following a dose-response gradient, with an ideal concentration of TGF-β1determined to be in the range of 50 to 100 ng/ml. No increments were observed with concentrations higher than 100 ng/ml, suggesting a ceiling effect.

The ideal platelet concentration for activated and non-activated PRP, regarding the potential to induce mesenchymal cell proliferation, was addressed by Wang et al. (2018a) Proliferation cell was increasingly more pronounced for platelet concentration from 200.000/ml to 1.500.000/ml, but with no further increments above 1.500.000/ml, which reinforces the existence of a ceiling effect.

##### Commercial kits for PRP preparation

Four studies compared the performance of different commercial kits, regarding platelet, leukocytes and GF concentration and platelet activation (Castillo et al., 2011; Magalon et al., 2014; Degen et al., 2017; Fitzpatrick et al., 2017).

Castillo et al. (2011) compared three commercial kits for PRP preparation (MTF Cascade, Arteriocyte Magellan, and Biomet GPS III PRP), in relation to platelet, leukocyte concentrations and to the concentration of PDGF-AB, PDGF-BB, TGF-β1, and VEGF. There was no statistically significant difference in relation to platelet concentration across different types of commercial kits. However, leukocyte concentration was significantly lower for the MTF Cascade system, followed by the Arteriocyte Magellan system. PRP produced by the Biomet GPS III system presented the highest leukocyte concentrations. There were observed differences related to the concentration of PDGF-AB, PDGF-BB, and VEGF, with no differences in TGF-β1 concentration. Arteriocyte Magellan yielded PRP with statistically higher concentrations of PDGF-AB and PDGF-BB, when compared to MTF Cascade. PRP produced by Biomet GPS III presented the highest concentration of VEGF.

The performance of several systems was assessed by Degen et al. Commercial kits that were tested included Arteriocyte Magellan, Biomet GPS III, Arthrex Angel 2% and 7%, Emcyte Genesis CS and Harvest SmartPrep APC + (Degen et al., 2017). Centrifugation protocols varied across different commercial kits, respecting the recommendations of manufacturers. Outcomes assessed included platelet and leukocyte concentration and pH. Overall, there was no significant differences related to platelet concentration, except for the 7% Arthrex Angel system, which led to higher concentrations of platelets than Genesis CS (2,310,000 ± 524,000 vs. 1,129,000 ± 264,000/mm^3^). In relation to leukocyte concentration, the observed variability did not reach statistical significance, with the exception of 2% Arthrex Angel, that showed statistically significant differences when compared to GPS III (11,000 ± 4,500 vs 27,300 ± 7,100/mm^3^). The pH of PRP obtained with SmartPrep APC + was lower (6.95 ± 0.06) when compared to other systems (≥ 7.26 ± 0.06).

Fitzpatrick compared four commercial systems for PRP production (PS III, Smart-Prep2, Arteriocyte Magellan, and ACP), in relation to platelet and leukocyte concentration, pH and platelet activation (Fitzpatrick et al., 2017). ACP system was associated with lower platelet concentration (1 to 1.7 times basal values), when compared to the PS III, Smart-Prep2 and Arteriocyte Magellan systems, which were associated to increases in platelet concentration in the magnitude of 3 to 6 times of basal values. The only system associated with leukocyte reduction was the ACP system (1,300/mm^3^; reduction of 5 to 22 times the basal values). The other systems were associated with increases in the concentration of leukocytes from 3 to 5 times the basal values. Mean pH of end product ranged between 6.59 (SmartPrep) to 7.05 (GPS). Lower levels of pH were associated with ACD-A.

In the study by Magalon et al., five systems were compared, two using a gel separation (SelphylSystem e RegenPRP), and three using centrifugation (Mini GPS III, Arthrex ACP, and the system developed in the laboratory study) (Magalon et al., 2014). Outcomes assessed included platelet, leukocyte and GF (VEGF, PDGF-AB, EGF, and TGF-b1) concentrations, and platelet activation. Mini GPS III System yielded higher platelet concentrations, when compared with the laboratory system, which by its turn was associated with higher platelet concentrations than the Regen PRP and Selphyl Systems. Mini GPS III and Regen PRP systems produced PRP with leukocyte concentration, as oppose to the Selphyl System, and the laboratory system, which led to leukocyte concentrations lower than basal values. Mini GPS III System was associated with higher concentrations of VEGF and EGF.

##### Anticoagulant and antiaggregating agents employed during PRP preparation

Amaral et al. (2016) compared PRP obtained with different anticoagulants regarding the potential of inducing proliferation of mesenchymal cells. Anticoagulants employed were EDTA, sodium citrate and ACD-A. Outcomes assessed were platelet and GF (TGF-1 and VEGF) concentrations, mean platelet volume. PRP generated from blood samples collected with EDTA exhibited more platelets, followed by sodium citrate and ACD-A. The number of platelet cells obtained with sodium citrate was 16,3% lower in relation to the EDTA samples, while the number of platelets in ACD-A samples was 23% lower than EDTA and 8% lower than sodium citrate. However, mean platelet volume was higher in EDTA samples, which suggests alterations in platelet morphology and reduced cell viability. Despite these findings, no difference in relation to TGF-1 and VEGF concentrations. Sodium citrate samples were associated with less proliferation of mesenchymal cells.

Two protocols for PRP production were compared by Anitua et al., one called physiological protocol, with less anticoagulants (0.4 mL of trisodium citrate 3,8%) and less intense platelet activation to a conventional protocol (0,9 mL of trisodium citrate 3,8%) (Anitua et al., 2016). Therefore, two interventions were simultaneously applied, preventing estimates for each intervention in separate. Assessed outcomes were platelet concentration, platelet activation, GF concentration (TGFβ1, IGF-1, VEGF, and PDGF-AB), and induction of fibroblast proliferation. Physiological protocol was associated with higher platelet and GF concentration, although exhibiting less platelet activation.

The employ of anticoagulants (ACD-A or heparine), in isolation or combined to the antiaggregant PGE1, was assessed by Fukaya and Ito (2014) Two activation methods were employed (0,5% Triton X and calcium gluconate 8.5%), beyond a control group with no activation. Assessed outcomes included platelet and PDGF-BB concentrations. In relation to platelet concentration, results were inconsistent across different samples, preventing conclusions. Both for inactivated and calcium-gluconate activated PRP, higher PDGF-BB concentrations were obtained with the concomitant utilization of ACD-A and PGE1. Results for PRP activated by Triton X were inconsistent across tested samples.

Kraus et al. (2018) compared ACD-A to sodium citrate, in relation to platelet concentration and morphology, through automatized analysis. The utilization of ACD-A was associated with higher platelet concentration, as well as with evidence of a more intense platelet activation, when compared to the employ of sodium citrate.

Other study compared three different types of EDTA, sodium citrate and ACD-A during PRP production for alopecia treatment in males (Singh, 2018). Platelet concentration and morphology were assessed, however clinical outcomes were not reported. Platelet concentration was higher in ACD-A samples (310%), when compared to EDTA (110%), or sodium citrate (100%) (p < 0,001). Morphological aspects, such as size, shape and the activation pattern, were more preserved in ACD-A samples.

##### Methods for activation

Lachert et al. (2011) compared GF concentration in PRP and in platelet gel, before and after thawing. Platelet gel was obtained by activating PRP with thrombin solution. TGF β1 concentration was 7 to 9 times higher in platelet gel. Higher concentrations were obtained after thawing. The same finding was observed in relation to PDGF-AB concentrations.

Lee et al. (2013) compared inactivated PRP to PRP activated by lyophilized thrombin plus calcium chloride. Assessed outcomes were GF (PDGF-AB, PDGF-BB and TGF-β) concentrations. There were no statistically significant differences related to GF concentrations between activated and inactivated PRP.

Vahabi et al. (2017) compared the effects of PRP activated by 10% calcium gluconate to inactivated PRP, in relation to the potential to induce fibroblasts and osteoblasts proliferation *in vitro*. Activated PRP was associated with more intense cell proliferation, with statistically significant results.

In the study by Anitua et al. (2016) two different protocols for PRP production were compared, namely the physiological protocol, employing lower quantities of anticoagulants and activators, and the conventional protocol. Due to the combined employment of interventions, separate estimates for each intervention were not possible. PGRF-Endoret was the activation substance in both study arms, with varying concentrations (20 microl/ml in the physiological protocol and 50 microl/ml in the conventional protocol). The physiological protocol was associated with higher platelet and GF concentrations, but with lower platelet activation.

Sadeghi-Ataabadi et al. (2017) compared different concentrations of calcium chloride (2.5; 5 and 10%) to activate PRP. Authors assessed the properties of the fibrine matrix and the potential to induce fibroblast proliferation. Higher rates of cell adhesion and cell proliferation were obtained with 2.5% calcium chloride. Cultures with PRP activated by 10% calcium chloride presented cells with fusiform morphology and a parallel configuration of stress fibers, while cultures with PRP activated with lower concentrations of calcium chloride showed typical fibroblast cells and stress fibers distributed in a net configuration.

Cavallo et al. (2016) compared 10% calcium chloride, 10% autologous thrombin, calcium chloride plus autologous thrombin, or 10% type I collagen. Assessed outcomes included concentrations of VEGF, TGF-β1 and PDGF-AB. Activation by collagen type I was associated with an overall reduction of GF concentrations. PRP activated by thrombin, calcium chloride plus autologous thrombin, and 10% type I collagen showed an immediate release of PDGF, and a progressive pattern of VEGF release along the period from 15 min to 24 h. Calcium chloride was associated with a progressive release of all GFs, with release starting from 15 min after activation up to 24 h.

Çetinkaya et al. (2016) compared activation by freezing to 10% calcium gluconate. Freezing temperature was −80°C for 24 h. Assessed outcomes included IGF-1, PDGF-BB, and βFGF concentrations. There was statistically significant difference related to PDGF concentration, favoring activation by freezing. There were no statistically significant differences concerning other GFs.

Thermal methods of activation were compared to activation with thrombin in the study by Du et al. (2018). The thermal protocol consisted in centrifugation under 4oC, with subsequent reheating to 37oC. Assessed outcomes included platelet count, GF (VEGF, PDGF-AB, PDGF-BB, TGF-α, βFGF, EGF and IGF) concentration. Platelet concentration was significantly higher with PRP activated by thermal methods. The pattern of GF release was considered more stable in samples activated with the thermal protocol.

Tunali et al. (2014) compared activation by the employ of titanium tubes to PRP with no activation, having assessed the histological properties of the fibrin net. Titanium activation was associated with larger fibrin nets.

Two studies compared the effects of activated and non-activated PRP on clinical outcomes. In the study conducted by Gentile et al. (2017) one group of participants with androgenetic alopecia were treated with autologous non-activated PRP and the other group was treated with calcium-activated PRP. Both activated and non-activated PRP groups presented increases in epidermal thickness and number of follicles, but concentrations of PDGF-BB, TGF-β1, and VEGF were higher in activated PRP. In Gentile et al. (2020), participants with androgenetic alopecia received activated and non-activated PRP. Short-term results in trichoscopy with non-activated PRP were more expressive than those observed in the activated-PRP group. This difference was statistically significant (*p* < 0.01). Long-term results of hair density also favored non-activated PRP (Gentile and Garcovich, 2020a).

##### Centrifugation protocols

###### Single versus double centrifugation

Single centrifugation protocol was compared to double centrifugation for PRP production in the study by Carofino et al. (2012). Single centrifugation protocol consisted in centrifugation under 1,500 rpm for 5 min. Double centrifugation involved a first centrifugation under 1,500 rpm for 5 min, followed by a second centrifugation under 6,300 rpm for 20 min. Centrifugal force was not reported. Assessed outcomes included platelet and leukocyte concentrations. Single protocol resulted in platelet concentration 3.6 times the basal values, while the double protocol resulted in increases of 3.3 times the basal values. Double centrifugation protocol was associated with lower leukocyte concentrations.

Mazzocca et al. (2012) compared three centrifugation protocols, in relation to platelet, leukocyte and GF (VEGF, HGF, IGF-1 and PDGF-AB) concentration and in relation to the potential to induce proliferation of human bone and muscle cells. Protocol 1 consisted in a single centrifugation under 500 rpm for 5 min. Protocol 2 involved a single centrifugation under 3200 rpm for 15 min. Protocol 3 involved two centrifugations, the first under 1500 rpm for 5 min, and the one under 6,300 rpm for 20 min. Centrifugal forces were not reported.

Protocol 2 was associated with higher platelet counts when compared to protocols 1 and 3. There was no statistically significant differences between protocols 1 and 3. Protocol 2 also resulted in the highest leukocyte counts (20,500 ± 6,700/mm^3^), while Protocol 1resulted in lowest leukocyte counts (600 ± 300/mm^3^). Protocol 3 resulted in intermediate values for leukocyte counts (1,700 ± 1,800/mm^3^), with values lower than those in whole blood (5,600 ± 1,700/mm^3^). Protocol 2 was the most effective in obtaining higher GF concentrations, with exception of VEGF-A. Protocol 1 was associated to higher concentrations of HGF, IGF-1 and PDGF-AB, in comparison to Protocol 3. Protocol 3 was more effective in inducing osteoblast proliferation, with no differences between Protocols 1 and 2. There was no difference across the three protocols in relation to myocyte or tenocyte proliferation.

Pochini et al. compared a single centrifugation protocol to two commercial kits employing double centrifugation, namely the Magellan and the GPSIII systems, in relation to platelet, leukocyte and GF (FGF-2 e TGF-beta1) concentrations (de Pochini et al., 2016). Both systems are associated with high leukocyte concentrations in the end product. The single centrifugation protocol consisted in applying a centrifugal force of 650 *g* for 8 min. The protocols of double centrifugation were performed as recommended by each manufacturer. The single-centrifugation protocol was associated with higher concentrations of TGF-β1, but with a lower concentration of FGF-2, when compared to both double-centrifugation protocols. The platelet concentrations obtained by the employ of the Magellan system was 2.7 (CI95% 2.11-3.95) times higher than those of samples processed with the GPSIII system. PRP obtained by the employ of the single-centrifugation protocol presented the lowest platelet concentrations. The GPSIII system was associated with the highest leukocyte concentrations, followed by the Magellan system.

Tamimi et al. (2007) compared a single-centrifugation protocol to double centrifugation for the prepare of PRP gel (Tamimi et al., 2007). Single centrifugation protocol consisted in applying 280g (1500 rpm) for seven minutes. The double centrifugation protocol consisted in applying 160 *g* (1300 rpm) for 10 min during the first centrifugation, followed by a second centrifugation of 400 *g* for 10 min. Platelet count and the ultrastructural analysis of PRP gel were assessed. Higher platelet concentrations were observed with the employ of the double-centrifugation protocol (352% of basal values), in comparison with single centrifugation (232% of basal values). However, the double-centrifugation protocol was associated with ultrastructure alterations of PRP gel, with fibrin agglutination.

Three centrifugation protocols for PRP processing were compared in the study by Kutlu et al. (2013) Protocol 1 employed single centrifugation at 43 *g* (1000 rpm) for 10 min. Protocol 2 employed double centrifugation, with the first at 103 g (2400 rpm) for 10 minutes and the second at 230 *g* (3600 rpm) for 15 min. Protocol 3 employed a first centrifugation at 129 *g* (3000 rpm) for three minutes and the second at 129 *g* (3000 rpm) for 13 min. PRP obtained by double-centrifugation protocols (Protocols 2 and 3) were associated with higher platelet concentrations. There was no statistically significant difference between Protocol 2 and 3.

###### Centrifugal forces

Kececi et al. (2014) employed a double-centrifugation protocol, by varying the centrifugal forces during the second centrifugation. The first centrifugation was performed at 250 *g* for 10 min. The second centrifugation was performed at 300, 500, 750, 1000, 1500, and 2000 *g* for 10 min. Platelet concentrations increased as centrifugal forces raised from 300 to 2000 *g*. The magnitude of increases was of 1.92, 2.16, 2.80, 3.48, 3.67, and 3.76 times basal values for centrifugal forces of 300, 500, 750, 1000, 1500, and 2000 *g*, respectively.

Ehrenfest et al. compared four commercial centrifuges (original L-PRF centrifuge^®^, A-PRF 12^®^, Salvin 1310^®^ and LW -UPD8^®^ for the processing of L-PRF (Ehrenfest et al., 2018). All samples were centrifuged once at 400 *g* for 12 min. Cell morphology and features of the fibrin matrix were assessed. The PRF obtained with the Intra-Spin^®^ centrifuge showed a highly polymerized fibrin matrix, with thick fibrin fibers and cells presenting physiological morphology. The other centrifuges produced PRF with thinner fibrin fibers, and irregular body cells with reduced dimensions.

In the study by Perez et al. (2014) the effects of varying centrifugal forces in both stages of double-centrifugation protocols were investigated. First centrifugation applied centrifugal forces ranging from 50 to 820 *g* (50, 70, 100, 190, 280, 370, 460, 550, and 820) for 10 min. The second centrifugation applied forces of 200, 400, 800, 1200, and 1600 *g* for 10 min, after a standard first centrifugation at 100 *g* for 10 min. Authors assessed platelet concentrations and platelet integrity. For the first centrifugation, greatest platelet concentrations were observed between 70 to 100 *g*, with decreases being observed above 190 *g*. The recovery rate of leukocytes ranged between 5 to 10%, independently of the centrifugal force applied during the second centrifugation. The most effective protocol for optimizing platelet concentration (5 times the basal values) was the double-centrifugation protocol, at 100 *g* for 10 min during the first centrifugation, followed by a second centrifugation at 400 *g* for 10 min. This protocol was also associated with platelet integrity.

###### Duration of centrifugation

Eren et al. (2016) compared 10 to 12-minute centrifugation for PRF processing, in relation to GF concentrations and cell composition of the end product. A single-centrifugation protocol at 400 *g* (2660 rpm) for 10 or 12 min was applied. The analyses carried out at 24 and 72 h showed higher concentration of VEGF, in samples obtained with the 12-min centrifugation protocol. The duration of the centrifugation did not influence the concentration of PDGF and TGF-ß or the platelet concentration.

Yin et al. compared double-centrifugation protocols, by applying different durations and forces of centrifugation in both stages (Yin et al., 2017). The assessed outcomes included platelet function and the potential to induce proliferation of mesenchymal cells. First centrifugations were performed at 10 *g* for 15min; 110 *g* for 15min; 130 *g* for 10 min; 130 *g* for 15 min; 160 *g* for 10 min; 160 *g* for 15 min; or 180 *g* for 10 min. Second centrifugation was performed at 80 *g* for 10 min; 180 *g* for 15 min; 250 *g* for 10 min; 250 *g* for 15 min; 450 *g* for 10 min; or 450 *g* for 15 min. Results indicated that a first centrifugation at 160 *g* for 10 min, followed by a second centrifugation at 250 *g* for 15 min led to the highest platelet and GF concentration, with preservation of the platelet function (*P* < 0.05). PRP obtained under these conditions induced more proliferation and migration of mesenchymal cells (*P* < 0.05), but with no impact over cell survival.

###### Duration of PRP processing time

Abu Kasim and Al-Hassan (2016) compared processing times for PRP produced at room temperature. PRP obtained with an 8-h processing time was compared to samples processed along 24 h. Platelet and leukocyte counts, platelet activation, and pH were assessed. PRP prepared along the 24-h period exhibited lower leukocyte concentrations. Differences in pH were observed, with lower pH for samples prepared in 24 h (pH = 7.3 ± 0,05), when compared to 8-hour samples (pH = 7,4 ± 0,13) (*p* < 0.001). Authors concluded that the differences were not clinically relevant, however no microbiological testing was performed to guarantee lack of contamination.

###### Storage conditions

The utilization of residual plasma at 20 to 24°C after centrifugation with glucose additive solution at room temperature for storing PRP was evaluated in the study by Alhumaidan et al. (2011). Authors assessed platelet and leukocyte counts, and platelet morphology. There were no differences related to platelet or leukocyte counts. Samples stored in the glucose additive solution presented more physiological morphology, when compared to the storage in residual plasma.

###### Combined use of PRP and other substances

Carofino et al. (2012) assessed the utilization of PRP in isolation or simultaneously to lidocaine 1%, bupivacaine 0.5%, and methylprednisolone, in relation to the potential to induce tenocyte proliferation (Carofino et al., 2012). All three substances resulted in less tenocyte proliferation (*p* = 0,05), with more pronounced reductions observed lidocaine and bupivacaine.

The influence of two types of iodinated contrast on the PRP characteristics were assessed in one study, considering that iodinated contrasts are frequently employed to guide intra-articular application of PRP (Dallaudiere et al., 2018). Assessed outcomes were platelet concentration, percentage of platelet aggregation, and platelet activation. Iodinated contrasts employed were Iodixanol and Iopamidol. There were no differences between PRP in isolation to PRP in association with both types of contrasts.

The association of PRP to hyaluronic acid in relation to final TGF-b1 and PDGF-AA concentration was assessed in another study. Release of TGF-b1 and PDGF-AA on the fifth day were greater with PRP combined to hyaluronic acid.

Most important results are synthesized in Supplementary Table 2.

## Discussion

Our results reflect the great variability embed in each step necessary for the preparation of PRP and related products, from the choice of anticoagulants during blood collection to the use of activation methods.

Studied anticoagulants included EDTA, ACD-A and sodium citrate. The employ of ACD-A was associated to the preservation of platelet morphology, with no effects on GF concentrations. Sodium citrate was associated with greater induction of proliferation of mesenchymal cells.

Double-centrifugation protocols was associated with higher platelet concentrations and to the decrease of leukocyte concentrations. However, these protocols are associated with lower concentrations of GFs, such as HGF, IGF-1 and PDGF-AB, probably by the loss of GFs contained within leukocytes. For PRP gel, double-centrifugation protocols lead to ultrastructural alterations of the fibrin net and fibrin agglutination.

The duration of the centrifugation time has also been shown to influence the concentration of at least some GFs. Centrifugation at 400 *g* for 12 min seems to be superior to 400 *g* for 10 min, regarding the concentration of VEGF. The same was observed in relation to the centrifugal force. For double-centrifugation protocols, the optimal centrifugal force for the first centrifugation seems to range between 70 to 100 *g*. For the second step, centrifugation at 2000 *g* for 10 min result in a platelet concentration 3.76 times greater than the basal values. When platelet integrity and viability were considered, the optimal centrifugation protocol was at 100 *g* for 10 min for the first centrifugation, followed by a second centrifugation at 400 *g* for 10 min.

Commercial kits currently available for PRP preparation employ different protocols of centrifugation, and therefore, variability in the characteristics of the end product are expected. Indeed, the platelet concentration ranged from 1.7 to 6 times the basal values, across kits from different manufacturers. In relation to the time of PRP processing, one study compared 24-h to 8-h processing time. No differences were observed in relation to platelet concentrations; however, microbiological tests were not performed to ensure the safety of extending the processing time.

Activation of PRP by calcium gluconate 10% was associated with greater potential of inducing osteoblast and fibroblast proliferation, but not to higher platelet or GF concentrations in some studies. Thermal activation seems to be a viable alternative, being associated with higher platelet concentrations, when compared to the activation by calcium gluconate 10%. For PRF processing, the employ of titanium tubes as an activation method was associated to more extensive fibrin net.

Concomitant application of PRP to lidocaine, bupivacaine and methylprednisolone was found to impact the expected biological action of PRP, therefore, caution should be taken when considering the combined use of these substances. The same was not observed with iodinated contrasts, commonly used to guide intra-articular injections, or with hyaluronic acid, that may even have a synergic effect, increasing GF release.

All this variability in PRP processing imposes one further question, related to definition of the ideal characteristics of PRP, in terms of the optimal platelet and GF concentrations. Some of the included studies point to a dose-response effect between the platelet and GF concentrations and the expected biological effects of PRP, with a ceiling effect.

To the best of our knowledge, this is the first attempt to review comparative studies that focused on different methods for each stage of PRP processing. In the systematic review conducted by Gentile et al. focusing on optimal concentration of PRP, results shown that higher concentrations of PRP may be associated with a significant decline in cell proliferation (Gentile and Garcovich, 2020b), which stresses the need for standardization of procedures in this regard. In other systematic review, recently conducted by Chahla et al. (2017) studies in which PRP was used for musculoskeletal conditions were assessed in relation to the reporting of the applied methods for PRP processing or of the composition of the final product. Authors found that only 10% of studies provided a clear description of the preparation protocol and only 16% provided quantitative parameters on the final composition of PRP.

The major limitation of the present study refers to the need of analyzing each step of the production process independently. We acknowledge that PRP production is a sequential process, rather than a combination of independent steps, but a framework to explore all types of results presented in included studies was needed. As most included studies assessed a single step in the process rather than sequential processes, the applied framework was built to reflect how comparative research in the field is being developed. Our scoping review did not embrace all sources of diversity related to the preparation and administration of platelet-rich plasma, but the reason for this was the fact that we did not identify any study comparing different techniques for certain steps of the production process. As our study included only comparative research, it was not possible to present evidence or draw conclusions on questions such as the use of handmade techniques in comparison to use of commercial kits, or the effects of light activation versus other methods of activation. Similarly, we did not find comparative research on the influence of red blood cell or peripheral blood mononuclear cells or on the role of image guidance during the application of platelet concentrates.

Protocols for PRP production should be clearly defined for each stage of processing, in accordance with desired biological effects. All studies included in this review focused on laboratorial outcomes, such as platelet, leukocyte, and GF concentrations, or on the potential to stimulate cell proliferation. The choice of this type of outcome relies on feasibility issues, however, the lack of clinical trials comparing PRP obtained from different methods precludes ultimate conclusions about the definition of best methods for PRP processing, under the perspective of efficacy, effectiveness and safety. This conundrum becomes even more complex, considering the vast universe of clinical situations for which PRP has been used. It is logical to assume that the ideal characteristics of PRP should differ in relation to platelet, leukocyte and GF concentration, for each type of clinical situation.

## Conclusion

Evidences found in this scoping review showed great variability related to methods for different stages of PRP processing, such as choice of anticoagulants during blood collect, centrifugation protocols, employ of activation methods, among others. This variability may justify the variability of clinical effects of PRP across different clinical trials.

## Author Contributions

DP, ÂB, and RR developed the study protocol, performed searches, screened references, performed narrative synthesis, and developed the first draft. AA and AM-J participated in the protocol elaboration and revised the manuscript. All authors contributed to the article and approved the submitted version.

## Conflict of Interest

The authors declare that the research was conducted in the absence of any commercial or financial relationships that could be construed as a potential conflict of interest.
