# Supplementary material for: Technical Procedures for Preparation and Administration of Platelet-Rich Plasma and Related Products: A Scoping Review
Source: Front Cell Dev Biol. 2020 Dec 11;8:598816. doi: 10.3389/fcell.2020.598816 (PMC7759516; doi:10.3389/fcell.2020.598816)
Supplement: Supplementary file 1 [file Table_1.DOCX]

Supplementary Material

# Search Strategies

**MEDLINE via PubMed**

#1 ("Platelet-Rich Plasma"[Mesh] OR “platelet-rich plasma” OR “Plasma, Platelet-Rich” OR “Platelet Rich Plasma” OR "Platelet-Rich Fibrin"[MeSh] OR “Fibrin, Platelet-Rich” OR “Platelet Rich Fibrin” OR “L-PRF” OR “Leukocyte- and Platelet-Rich Fibrin” OR “Leukocyte and Platelet Rich Fibrin” OR “Platelet Rich Concentrate”)

#2 (Process*[Title/Abstract] OR Proceeding[Title/Abstract] OR Procedure[Title/Abstract] OR Manipulat*[Title/Abstract] OR “Standard operating procedure”[Title/Abstract] OR SOP[Title/Abstract] OR Execution[Title/Abstract] OR Obtain*[Title/Abstract] OR Way[Title/Abstract])

#3 #1 AND #2

**LILACS via BVS (Biblioteca Virtual em Saúde)**

#1 MH:"Plasma Rico en Plaquetas" OR MH:"Platelet-Rich Plasma" OR MH:"Plasma Rico em Plaquetas" OR MH:A12.207.152.693.600$ OR MH:A12.207.270.695.600$ OR MH:A15.145.693.600$ OR (Plasma Rico en Plaquetas) OR (Platelet-Rich Plasma) OR (Concentrado de Plaquetas)

#2 Processing OR Proceeding OR Procedure OR Manipulation OR (Standard operating procedure) OR Execution OR SOP OR Processamento OR Produção OR (Procedimento Operacional Padrão) OR Manipulação OR Execução OR Procesamiento OR Manipulación OR Producción

#3 #1 AND #2

**Embase via Elsevier**

#1 'thrombocyte rich plasma'/exp #2 'platelet-rich plasma'/exp #3 'platelet-rich plasma' or 'thrombocyte-rich plasma' #4 #1 or #2 or #3 #5 Processing OR Proceeding OR Procedure OR Manipulation OR 'Standard operating procedure' OR Execution OR SOP #6 #4 and #5 #7 #6 AND [embase]/lim NOT ([embase]/lim AND [medline]/lim)

# Excluded studies with reasons

| \| **Author** \| **Year** \| **Reasons for exclusion** \| \| --- \| --- \| --- \| \| Akhundov \| 2012 \| Non-comparative study \| \| Atefi \| 2015 \| Non-comparative study \| \| Beitzel \| 2015 \| Narrative review \| \| Bergamaschi \| 2013 \| Non-comparative study \| \| Chahla \| 2017 \| Systematic Review \| \| Chandler \| 2013 \| Non-comparative study \| \| Christensen \| 2006 \| Transfusion use \| \| Cioffi \| 2011 \| Non-comparative study \| \| de Melo \| 2018 \| Non-comparative study \| \| Dhurat \| 2014 \| Narrative review \| \| Eppley \| 2004 \| Non-comparative study \| \| Felthaus \| 2017 \| Non-comparative study \| \| Fiorentino \| 2015 \| Narrative review \| \| Fox \| 2018 \| Participants under antiaggregation therapy \| \| Franco \| 2012 \| Non-comparative study \| \| Giannini \| 2015 \| Comparative study with no statistical analyses \| \| Gómez \| 2015 \| Non-comparative study \| \| Gonshor \| 2002 \| Non-comparative study \| \| Gorokhova \| 2015 \| Non-comparative study \| \| Greening \| 2011 \| Non-comparative study \| \| Kaux \| 2011 \| Language (French) \| \| Kesi \| 2014 \| Language (Polish) \| \| Konokhova \| 2016 \| Non-comparative study \| \| Lorente-Pérez-Sierra \| 2011 \| Non-comparative study \| \| Lourenço \| 2018 \| Non-comparative study \| \| Lozano \| 2003 \| Non-comparative study \| \| Lucarelli \| 2010 \| Non-comparative study \| \| Magalon \| 2016 \| Non-comparative study \| \| Marques \| 2014 \| Non-comparative study \| \| Marques \| 2015 \| Narrative review \| \| Mazzzanti \| 2015 \| Non-comparative study \| \| Mijovic \| 2011 \| Non-comparative study \| \| Murphy \| 2015 \| Narrative review \| \| Nardi \| 2011 \| Additional publication of Merolla 2012 \| \| Nasiri \| 2015 \| Narrative review \| \| O'Connel \| 2012 \| Non-comparative study \| \| Ogundipe \| 2012 \| Non-comparative study \| \| Pajk \| 2011 \| Transfusion use \| \| Pereira \| 2012 \| Non-comparative study \| \| Plöderl \| 2012 \| Non-comparative study \| \| Plöderl \| 2011 \| Non-comparative study \| \| Rebulla \| 2014 \| Non-comparative study \| \| Rebulla \| 2016 \| Non-comparative study \| \| Rugg \| 2009 \| Wrong blood products as comparators \| \| Sheffeld \| 2009 \| Transfusion use \| \| Sheffeld \| 2009 \| Transfusion use \| \| Sonnlitner \| 2000 \| Non-comparative study \| \| Tsukao \| 2013 \| Animal model \| \| Varela \| 2018 \| Non-comparative study \| \| Vendramin \| 2009 \| Non-comparative study \| \| Wang \| 2013 \| Non-comparative study \| \| Wei \| 2014 \| Language (Chinese) \| \| Woodel-May \| 2005 \| Animal model \| \| Yang \| 2017 \| Language (Chinese) \| \| Yun \| 2015 \| Non-comparative study \| |
| --- | --- | --- | --- | --- | --- | --- | --- | --- | --- | --- | --- | --- | --- | --- | --- | --- | --- | --- | --- | --- | --- | --- | --- | --- | --- | --- | --- | --- | --- | --- | --- | --- | --- | --- | --- | --- | --- | --- | --- | --- | --- | --- | --- | --- | --- | --- | --- | --- | --- | --- | --- | --- | --- | --- | --- | --- | --- | --- | --- | --- | --- | --- | --- | --- | --- | --- | --- | --- | --- | --- | --- | --- | --- | --- | --- | --- | --- | --- | --- | --- | --- | --- | --- | --- | --- | --- | --- | --- | --- | --- | --- | --- | --- | --- | --- | --- | --- | --- | --- | --- | --- | --- | --- | --- | --- | --- | --- | --- | --- | --- | --- | --- | --- | --- | --- | --- | --- | --- | --- | --- | --- | --- | --- | --- | --- | --- | --- | --- | --- | --- | --- | --- | --- | --- | --- | --- | --- | --- | --- | --- | --- | --- | --- | --- | --- | --- | --- | --- | --- | --- | --- | --- | --- | --- | --- | --- | --- | --- | --- | --- | --- | --- | --- | --- | --- | --- | --- | --- |
