# Supplementary material for: Technical Procedures for Preparation and Administration of Platelet-Rich Plasma and Related Products: A Scoping Review
Source: Front Cell Dev Biol. 2020 Dec 11;8:598816. doi: 10.3389/fcell.2020.598816 (PMC7759516; doi:10.3389/fcell.2020.598816)
Supplement: Supplementary file 2 [file Table_2.DOCX]

**Table 2: Main results of included studies**

| Study | Intervention | Comparison | Outcomes | Main results |
| --- | --- | --- | --- | --- |
| Comparison: PRP compared to other platelet concentrates | | | | |
| Cavallo 2014 | PRP | L-PRP | Chondrocyte proliferation  GF concentration  Production of cartilage matrix | L-PRP associated with higher concentrations of GF.  PRP was associated with more expressive cell proliferation after seven days of cell culture. |
| Mariani 2015 | PRP | L-PRP | Antimicrobial properties | Both concentrates associated with inhibition of bacterial growth. |
| Kieb 2017 | PRP | PRP powder | Platelet concentration  Leukocyte concentration  GF concentration | PRP powder associated with higher concentrations of GF |
| Kobayashi 2015 | PRP | PRF | GF concentration  Neovascularization  Response to scratch assay | PRP associated with higher concentrations of PDGF  PRF associated with superior neovascularization and healing |
| Xian 2015 | 20% PRP | 10% PRP | GF concentration  Cell viability  Response to scratch assay | 10% PRP associated with higher concentrations of HGF and VEGF-a, more abundant keratinocyte proliferation.  20% PRP associated with more collagen fibers types I and III. |
|  |  |  |  |  |
| Comparison: PRP with different levels of GFs and platelet concentrations | | | | |
| Han 2007 | Different concentrations of TGF-β1 and PDGF compared to each other | | Proliferation of periodontal ligament cells | Ideal concentration of TGF-β1determined to be in the range of 50 to 100 ng/ml |
| Wang 2018 | Different platelet concentrations compared to each other | | Proliferation of mesenchymal cell | Ideal platelet concentration determined to be in the range of 200.000/ml to 1.500.000/ml |
| Comparison: Commercial kits for PRP preparation | | | | |
| Castillo 2011 | MTF Cascade, Arteriocyte Magellan, and Biomet GPS III PRP compared to each other | | Platelet concentration  Leukocyte concentration  GF concentration | Arteriocyte Magellan associated with higher concentrations of PDGF, when compared to MTF Cascade.  Biomet GPS III associated with the highest concentration of VEGF. |
| Degen 2017 | Arteriocyte Magellan, Biomet GPS III, Arthrex Angel 2% and 7%, Emcyte Genesis CS and Harvest SmartPrep APC+ compared to each other | | Platelet concentration  Leukocyte concentration | 7% Arthrex Angel system associated with higher platelet concentrations than Genesis CS  Biomet GPS III associated with higher leukocyte concentrations, when compared to 2% Arthrex Angel |
| Fitzpatrick 2017 | PS III, Smart-Prep2, Arteriocyte Magellan, and ACP compared to each other | | Platelet concentration  Leukocyte concentration | ACP system associated with lower platelet concentrations.  ACP system associated with leukocyte reduction. |
| Magalon 2014 | SelphylSystem, RegenPRP, Mini GPS III, Arthrex ACP, and owned system compared to each other | | Platelet concentration  Leukocyte concentration  GF concentration  Platelet activation | Platelet concentration: Mini GPS III System > owned system > Regen PRP and Selphyl Systems.  Leukocyte concentration: Mini GPS III and Regen PRP systems associated with leukocyte concentration. Selphyl System and owned system associated with leukocyte concentrations lower than basal values.  Mini GPS III System was associated with higher concentrations of VEGF and EGF. |
| Comparison: Anticoagulants and antiaggregating agents | | | | |
| Amaral 2016 | EDTA, sodium citrate and ACD-A compared to each other | | Platelet concentration  GF concentration  Platelet morphology and viability | Platelet concentration: EDTA > sodium citrate > ACD-A.  EDTA associated with alterations in platelet morphology and reduced cell viability. |
| Anitua 2016 | Physiological protocol (0.4 mL of trisodium citrate 3,8%) | Conventional protocol (0,9 mL of trisodium citrate 3,8%) | Platelet concentration  Platelet activation  GF concentration  Induction of fibroblast proliferation. | Physiological protocol associated with higher platelet and GF concentration and less platelet activation. |
| Fukaya 2014 | ACD-A or heparine, in isolation or combined to the antiaggregant PGE1 | | Platelet concentration  GF concentration | ACD-A + PGE1 associated with higher platelet concentrations. |
| Kraus 2018 | ACD-A | sodium citrate | Platelet concentration and morphology | ACD-A associated with higher platelet concentrations and more activation. |
| Singh 2018 | EDTA, sodium citrate and ACD-A | | Platelet concentration and morphology | ACD-A associated with higher platelet concentrations and more activation. |
| Comparison: Methods for activation | | | | |
| Lachert 2011 | Thawing | Conventional temperature | GF concentration | Thawing associated with higher GF concentrations. |
| Lee 2013 | Lyophilized thrombin plus calcium chloride | No activation | GF concentration | No statistically significant differences between groups. |
| Vahabi 2017 | 10% calcium gluconate | No activation | Induction of fibroblasts and osteoblasts proliferation | 10% calcium gluconate associated with more intense cell proliferation. |
| Anitua 2016 | PGRF-Endoret (20 microl/ml) | PGRF-Endoret (50 microl/ml) | Platelet concentration  GF concentration  Platelet activation | PGRF-Endoret (20 microl/ml) associated with higher platelet and GF concentrations and less platelet activation. |
| Sadeghi-Ataabadi  2017 | Different concentrations of calcium chloride (at 2.5; 5 and 10%), compared to each other | | Properties of fibrine matrix  Induction of fibroblasts proliferation | 2.5% calcium chloride associated with less intense cell proliferation and more physiological fibrine matrix. |
| Cavallo 2016 | 10% calcium chloride, 10% autologous thrombin, calcium chloride plus autologous thrombin, or 10% type I collagen | | GF concentration | Collagen type I associated with reduction of GF concentrations. |
| Çetinkaya 2016 | Freezing -80oC for 24 hours | 10% calcium gluconate | GF concentration | Freezing associated with higher PDGF concentrations. |
| Du 2018 | Thermal protocol (centrifugation under 4oC, with subsequent reheating to 37oC) | Thrombin | Platelet concentration  GF concentration | Thermal protocol associated with higher platelet concentrations. |
| Tunali 2014 | Use of titanium tubes | No activation | Properties of the fibrin net | Titanium activation associated with larger fibrin nets. |
| Gentili 2017 | Calcium activation | No activation | Epidermal thickness  Number of follicles  GF concentration | GF concentration higher with calcium activation. |
| Gentili 2020 | Calcium activation | No activation | Hair density | Non-activated PRP associated with higher hair density in the short- and long-term. |
| Comparison: Centrifugation protocol  Single versus double centrifugation | | | | |
| Carofino 2012 | Single centrifugation  (1,500 rpm for 5 minutes) | Double centrifugation  Cycle 1: 1,500 rpm for 5 minutes  Cycle 2: 6,300 rpm for 20 minutes. | Platelet concentration  Leukocyte concentration | Single centrifugation associated with higher platelet and leukocyte concentrations. |
| Mazzoca 2012 | Protocol 1: single centrifugation (500 rpm for 5 min).  Protocol 2: single centrifugation (3200 rpm for 15 min).  Protocol 3: two centrifugations (1500 rpm for 5 min + t6,300 rpm for 20 min). | | Platelet concentration  Leukocyte concentration  GF concentration  Induction of cell proliferation | Protocol 2 associated with higher platelet, leukocyte and GF concentrations.  Protocol 3 was more effective in inducing osteoblast proliferation. |
| Pochini  2016 | Single centrifugation  (650 g for 8 minutes) | Double centrifugation  (Magellan and GPSIII systems) | Platelet concentration  Leukocyte concentration  GF concentration | Platelet concentration: Magellan > GPSIII > single centrifugation  Leukocyte concentration: GPSIII > Magellan |
| Tamimi 2007 | Single centrifugation  (280g for 7 min) | Double centrifugation (160 g for 10 min + 400 g for 10 min) | Platelet concentration  Structural analysis of PRP gel | Double centrifugation associated with higher platelet concentrations.  Single centrifugation associated with preserved structure of PRP gel and less fibrin agglutination. |
| Kutlu 2013 | Protocol 1: single centrifugation (43 g for 10 min)  Protocol 2: double centrifugation (103 g for 10 min + 230 g for 15 min)  Protocol 3 double centrifugation (129 g for 3 min + 129 g for 13 min). | | Platelet concentration | Protocols 2 and 3 associated with higher platelet concentrations, with no statistically significant differences when compared to each other. |
| Comparison: Centrifugal forces | | | | |
| Kececi 2014 | First centrifugation: 250 g for 10 min  Second centrifugation: 300, 500, 750, 1000, 1500 and 2000 g for 10 min | | Platelet concentration | Platelet concentrations increased as centrifugal forces raised from 300 to 2000 g. |
| Dohan 2018 | Four commercial brands of centrifuges  Single centrifugation protocol (400 g for 12 minutes) | | Cell morphology  Structure of the fibrin matrix | Intra-Spin® centrifuge associated with more polymerized fibrin matrix and cells presenting physiological morphology. |
| Perez 2014 | First centrifugation ranging from 50 to 820 g (50, 70, 100, 190, 280, 370, 460, 550 and 820) for 10 min  Second centrifugation of 200, 400, 800, 1200 and 1600 g for 10 min, after a first centrifugation at 100 g for 10 min. | | Platelet concentration  Platelet morphology | First centrifugation: 70 to 100 g associated with higher platelet concentrations.  Protocol with higher platelet concentration was 100 g for 10 min + 400 g for 10 min. This protocol was also associated with platelet integrity. |
| Comparison: Duration of centrifugation | | | | |
| Eren 2016 | 10 min (400 *g*) | 12 min (400 *g*) | Platelet concentration  GF concentration | 12-min centrifugation was associated with higher VEGF concentration.  Protocols did not differ in relation to platelet concentration. |
| Yin 2017 | First centrifugations: 10 g for 15min; 110 g for 15min; 130 g for 10 min; 130 g for 15 min; 160 g for 10 min; 160 g for 15 min; or 180 g for 10 min.  Second centrifugation: 80 g for 10 min; 180 g for 15 min; 250 g for 10 min; 250 g for 15 min; 450 g for 10 min; or 450 g for 15 min. | | Platelet function  Induction of proliferation of mesenchymal cells | 160 g for 10 min + 250 g for 15 min associated with the highest platelet and GF concentration and more proliferation of mesenchymal cells. |

*ACD-A: anticoagulant Citrate Dextrose; EDTA: ethylenediamine tetraacetic acid; EGF: epidermal growth factor; L-PRP: leukocyte- and platelet-rich plasma; PDGF: platelet-derived growth factor; PGE1: prostaglandin E1; PRP: platelet-rich plasma; TGF-β1:* *transforming growth factor beta 1; VEGF: vascular endothelial growth factor*
